# MicroRNA Mediating Networks in Granulosa Cells Associated with Ovarian Follicular Development

**DOI:** 10.1155/2017/4585213

**Published:** 2017-02-19

**Authors:** Baoyun Zhang, Long Chen, Guangde Feng, Wei Xiang, Ke Zhang, Mingxing Chu, Pingqing Wang

**Affiliations:** ^1^Bioengineering Institute of Chongqing University, Chongqing, China; ^2^Sichuan TQLS Animal Husbandry Science and Technology Co., Ltd., Mianyang, China; ^3^Key Laboratory of Farm Animal Genetic Resources and Germplasm Innovation of Ministry of Agriculture, Institute of Animal Science, Chinese Academy of Agricultural Sciences, Beijing, China

## Abstract

Ovaries, which provide a place for follicular development and oocyte maturation, are important organs in female mammals. Follicular development is complicated physiological progress mediated by various regulatory factors including microRNAs (miRNAs). To demonstrate the role of miRNAs in follicular development, this study analyzed the expression patterns of miRNAs in granulosa cells through investigating three previous datasets generated by Illumina miRNA deep sequencing. Furthermore, via bioinformatic analyses, we dissected the associated functional networks of the observed significant miRNAs, in terms of interacting with signal pathways and transcription factors. During the growth and selection of dominant follicles, 15 dysregulated miRNAs and 139 associated pathways were screened out. In comparison of different styles of follicles, 7 commonly abundant miRNAs and 195 pathways, as well as 10 differentially expressed miRNAs and 117 pathways in dominant follicles in comparison with subordinate follicles, were collected. Furthermore, SMAD2 was identified as a hub factor in regulating follicular development. The regulation of miR-26a/b on* smad2* messenger RNA has been further testified by real time PCR. In conclusion, we established functional networks which play critical roles in follicular development including pivotal miRNAs, pathways, and transcription factors, which contributed to the further investigation about miRNAs associated with mammalian follicular development.

## 1. Introduction

The mammalian ovary is a dynamic organ. The coordination of follicle recruitment, selection, and ovulation and the timely development are essential for a functional ovary and fertility [1]. Follicular development is a highly accurate, orchestrated, and periodic process which starts with the activation of resting follicles gradually leading to the growth and selection of dominant follicles (DFs) from small health follicles accompanied with sequential and profound differentiation of oocyte and the surrounding somatic cells [2], especially granulosa cells (GCs) [3]. Understanding the molecular mechanism of follicular development is essential for unraveling the complex synergies orchestrated during the process of forming the fertilizable ovum. In an estrous cycle, small follicular growth is characterized by 2 or 3 successive follicular waves which coincide with the luteinizing hormone- (LH-) surge waves. During the waves, a single follicle is selected, normally the largest (occasionally the largest two), which continuously grows as a DF while the others, referring to subordinate follicles (SFs), terminate development and undergo atresia [4]. Both of the two kinds of follicles are named large healthy follicles. The complex transition from primordial follicles to mature follicles is due to the functional differentiation and morphological transformation of GCs. In this crucial period, the growth of oocyte depends on the bidirectional communication between oocyte and GCs [5, 6]. Hence, the proliferation, apoptosis, and remarkable functional differentiation of GCs are significant events and required for follicle maturation [7]. Each of these development steps involves significant changes of follicular structure and function, requiring accurate and coordinated adjustments to genes which have key roles in follicular selection, maturation, or the follicle-luteal transition. Any dysregulation in the expression of these specific genes would be critical in determining the fate of DFs or SFs [8–11]. In previous studies, transcriptome analyses have identified some genes involved in follicular growth, selection, and maturation [12–14]. However, the molecular regulatory mechanisms at differential levels are still unclear.

Recently, the posttranscriptional regulation dominated by miRNAs has attracted extensive attention. miRNAs regulate gene expression via the combination of seed sequence and the 3′-untranslated region (UTR) of target mRNAs, causing repression of translation or degradation of the target mRNAs during cell growth and differentiation [15]. The expression of miRNA in the ovary varies with cell type, function, and stage of the estrous cycle. miRNAs are involved in the formation of primordial follicles, follicular recruitment and selection, follicular atresia, oocyte-cumulus cell interaction, and GC function [1]. Profiling studies of miRNA in ovarian tissues have described the expression of miRNAs in the ovaries of various species [16–20]. Conditional knockout (cKO) of Dicer1 from follicular GCs resulted in a number of ovarian functional defects including abnormal oocyte maturation, disrupted follicular development and ovulation, increased follicular atresia, and infertility [21–23]. A single miRNA could regulate follicle development during estrus cycle via a canonical pathway [24], in which the target gene of miRNA plays an important role. Unsurprisingly, many miRNAs also could target transcription factors (TFs), such as TGF-*β* superfamily members, follicle stimulating hormone receptor (FSHR), and luteinizing hormone receptor (LHR), which have been confirmed to have a connection with follicular development. The abnormality of these molecules also led to dysfunction of cellular communication and dysregulation of normal follicle development and recruitment [25–29]. Moreover, several studies have demonstrated that the functions of specific miRNAs are implicated in different aspects of GC processes [30] such as proliferation [31–34], survival [35–37], terminal differentiation [38], steroidogenesis [31, 33, 39, 40], and cumulus expansion [41] in mammals. For example, miRNA-224 has been proved to be involved in transforming growth factor-beta-mediated mouse GCs proliferation and GC function by targeting* smad4* [31]. These results present evidence that miRNA might be involved in the selection of the DFs, the mechanism of which has remained largely elusive. The complex nature of miRNA target interaction, regulation, and function, however, posed challenges for functional studies. Whereas some miRNAs appear single-handedly to regulate specific signaling pathways, most miRNAs act in clusters and are fine tuners of cellular functions. In most of previous studies, results just demonstrated the function of a single miRNA in GCs. In fact, performing significant regulation of any biological process often results from complex regulating networks rather than one single miRNA. Therefore, an understanding of the mechanism of action of miRNA in follicular development requires global comprehension of the network of miRNA target interactions within the milieux of other factors that dominate follicular development. Hence, profiling studies are important in order to not only draw a spatial and temporal map of miRNA expression in different follicles, but also provide clues with regard to the function or regulation of miRNA.

In this study, according to 3 underused sequencing datasets (GSE56002, GSE55987, and GSE54692) from GCs of bovine, we attempted to systematically dissect the complex synergistic regulations of several functional miRNAs rather than a single miRNA in follicular development [42], based on the networks of miRNAs-signal pathways and miRNAs-TFs. Abundant miRNAs and differentially expressed miRNAs were identified and networks of miRNAs-signal pathways and miRNAs-TFs were constructed based on the correlation between miRNAs and predicted target genes. Moreover, by calculating the degree of every node in networks, hub players were identified to establish a centrical network, from which the critical miRNAs, pathways, and TFs in the process of follicular development were identified. The regulation of the significant miRNAs on hub genes would be further verified by RT PCR. The results might provide novel insights into revealing the potential mechanism of molecular regulation in follicular development in the context of miRNA synergistic regulatory networks.

## 2. Materials and Methods

### 2.1. Differential Expression of miRNAs in GCs and Data Normalization

A total of 64 differentially expressed miRNAs in GCs were collected from the miRNA sequencing results of Samuel Gebremedhn's research group (GSE56002) [24] and 52 ones from the study of another group, Salilew-Wondim et al. who also utilized Illumina small RNAseq (GSE55987) [47]. Gebremedhn et al. used miRDeep 2.0.0.5 software package and DESeq2 with a “hypothetical reference” to screen differentially expressed miRNAs in 6 ovarian follicle samples (3 biological replicates from DFs and others from SFs) at day 19 of the estrous cycle, in which the samples with external surface diameter ≥ 12 mm were recognized as DFs while the ones ≤ 11 mm as SFs. The study followed several cut-off criteria, adjusted* p* value ≤ 0.05, log2 fold change ≥ 1, and false discovery rate (FDR) ≤ 0.1 [24]. Similarly, Salilew-Wondim et al. employed the analogous software and methods to analyze the differential expression of miRNAs under the same criteria, which were obtained from 12 granulosa samples (three biological replicates of GCs from SFs or DFs at day 3 or day 7 of the estrous cycle) [47]. Briefly, in this study, follicles with follicular diameter of 6 mm (*n* = 43) were classified as SFs, while follicles with a diameter of 8–10 mm (*n* = 9) were considered as DFs at day 3 of the estrous cycle. On the other hand, at day 7 of the estrous cycle, follicles with a diameter ≤ 8 mm (*n* = 58) were considered as SFs while those with 9–13 mm diameter (*n* = 3) were categorized as DFs. Although there are some biological differences between day 3 and day 7, this did not exactly influence our analysis since what we considered was the whole process of DFs and SFs. By comparison, abundantly expressed or dysregulated miRNAs in both datasets were dissected in this study. Meanwhile, another dataset (GSE54692), which utilized microarray to assess differentially expressed miRNAs in GCs of bovine, was investigated in the same manner, and the miRNAs meeting the same criteria mentioned above were selected [48]. In summary, after comparison of small and DFs, we firstly obtained 17 dysregulated miRNAs from the two types of follicles. Then, 57 dysregulated miRNAs were identified between large atretic follicles and DFs. Finally, a total of 15 miRNAs which played key roles in both growth and selection phase of DFs were further detected and analyzed in this paper.

### 2.2. miRNA Target Gene Prediction and Relevant Functional Analysis by GO and Pathway Enrichment

There were no online databases to predict the target genes of miRNAs in bovine. In this context, through sequence alignment, we confirmed that there was homology of miRNAs between human and bovine, and since the prediction of target genes was based on algorithm of sequence, this study analyzed patterns on the regulating functions of miRNAs in bovine follicular development through their target prediction along with human species. The potential target genes of differentially expressed miRNAs in GCs were acquired from the widely used online databases TargetScanHuman 7.0 (http://www.targetscan.org/) [49] with conservation (aggregate *P*_CT_ > 0.8) and context score (<−0.4 and percentile > 85%) and DIANA-microT (http://diana.imis.athena-innovation.gr/DianaTools/index.php?r=microT_CDS/index) [50]. The aggregate *P*_CT_ is calculated as

(1) PCT=1−1−PCTsite11−PCTsite21−PCTsite3⋯.

In order to reduce false positives, the predicted target genes which appeared at both databases were accepted. Thus, the collection of predicted target genes of each obtained miRNA was imported into DIANA-mirPath (http://diana.imis.athena-innovation.gr/DianaTools/index.php?r=mirpath/index), a miRNA-pathway analysis web server. Each list of canonical pathways significantly affected by differentially expressed miRNAs was made a contrast with another list and we obtained the common pathways which were as targets of dysregulated miRNAs. All the following canonical pathways were identified from the Kyoto Encyclopaedia of Genes and Genomes (KEGG) databases.

As a comprehensive set of functional annotation tools, DAVID (the Database for Annotation, Visualization and Integrated Discovery) has been used for integrative and systematic analysis of enormous gene lists [51]. GO terms are significantly overrepresented in a set of genes from three aspects, namely, the biological process, cellular component, and molecular function. In this study, the key GO biological process terms of the predicted target genes of miRNAs were performed using DAVID with the thresholds of enrichment gene count > 2 and* p *value < 0.05.

### 2.3. miRNA-Pathway Network and miRNA-TF Network Construction

Study on pathways associated with dysregulated miRNAs was carried out using DIANA-mirPath. The results were integrated to get the intersection from those meeting* p* value threshold (Benjamini and Hochberg's FDR was applied with significant threshold set at* p* value ≤ 0.05) and microT threshold (0.8 of the score). Then the interactions between miRNA and pathways were collected to construct miRNA-pathway network. Transcription factors that can be involved in control of particular ovarian functions were screened from Animal Transcription Factor Database (http://www.bioguo.org/AnimalTFDB/BrowseAllTF.php?spe=Homo_sapiens) and Sirotkin's study [52]. In order to improve veracity, target genes of miRNAs were predicted via different software. TFs which might be regulated by miRNAs were also identified. Then the selected miRNAs-TFs database was used for constructing network by Cytoscape software under the similar protocol [53]. Furthermore, through calculating the degree of each node, hub TFs were selected to construct the core network.

### 2.4. Cell Culture and Transfection

A steroidogenic human granulosa-like tumor cell line (KGN) was undifferentiated and maintained the physiological characteristics of ovarian cells. The KGN cells were cultured in DMEM basic (1x)/high glucose medium (Gibco, Life Technologies, Carlsbad, CA, USA) containing 12% fetal bovine serum (FBS; Gibco, Australia) and 1% antibiotics (100 U/ml penicillin and 100 *μ*g/ml streptomycin; Sigma) in a humidified incubator at 37.0°C with 5% CO_2_. For human granulosa cells culture, the GCs were first purified as described previously [54]. GCs were cultured at a final concentration of 5 × 10^5^ viable cells/ml culture medium, in 12-well plate at 37.0°C and 5% CO_2_. After 24 h, the medium was replaced with RPMI only; then 4 *μ*g miR-26a/b mimics, inhibitors, or miRNA expression vectors pSUPER-miR-26a/b [55] (with a negative control) were transfected using Lipofectamine 2000. The sequence of miR-26a/b mimics and miR-26a/b inhibitors, as well as their negative controls (Genepharma, Shanghai, China), are as follows:

  miR-26a mimics: 5′-UUCAAGUAAUCCAGGAUAGGCU-3′
  miR-26a inhibitors: 5′-AGCCUAUCCUGGAUUACUUGAA-3′
  miR-26b mimics: 5′-UUCAAGUAAUUCAGGAUAGGU-3′
  miR-26a inhibitors: 5′-ACCUAUCCUGAAUUACUUGAA-3′
  Mimics negative control: 5′-UUCUCCGAACGUGUCACGUTT-3′
  Inhibitors negative control: 5′-CAGUACUUUUGUGUAGUACAA-3′

After 8 h, the cells were subsequently treated with complete medium followed by extraction of total RNA from the cultured cells using Trizol (TaKaRa) at 48 h later.

### 2.5. Real Time PCR Assay

For RT PCR assays, the cDNA was synthesized from 1000 ng of purified RNA using the PrimeScript RT reagent kit (TaKaRa Bio, Inc., Otsu, Japan) following the manufacturer's instructions. RT PCR was performed in an Applied Biosystems Step One RT PCR system using a SYBR Premix Ex Taq II Kit (Takara Bio, Inc., Shiga, Japan) and RT PCR machine (Bio-Rad C1000PCR, USA). Each sample was analyzed in triplicate and the experiment was repeated three times. The primers for* smad2*, miR-26a, miR-26b, U6, and *β-actin* are designed as follows:

 
*smad2* forward: 5′-AGAAGCAGCTCGCCAGCCAG-3′
 
*smad2* reverse: 5′-CGGCGTGAATGGCAAGATGG-3′
  miR-26a stem-loop primer: 5′-CTCAACTGGTGTCGTGGAGTCGGCAATTCAGTTGAGAGCCTATC-3′
  miR-26a forward: 5′-ACACTCCAGCTGGGTTCAAGTAATCCAGGA-3′
  miR-26b stem-loop primer: 5′-CTCAACTGGTGTCGTGGAGTCGGCAATTCAGTTGAGACCTATCC-3′
  miR-26b forward: 5′-ACACTCCAGCTGGGTTCAAGTAATTCAGG-3′
  Universal reverse primer: 5′-TGGTGTCGTGGAGTCG-3′
 
*β-Actin* forward: 5′-AAAGACCTGTACGCCAACAC-3′
 
*β-Actin* reverse: 5′-GTCATACTCCTGCTTGCTGAT-3′
  U6 forward: 5′-CTCGCTTCGGCAGCACA-3′
  U6 reverse: 5′-AACGCTTCACGAATTTGCGT-3′

PCR conditions were set as follows: 95°C for 30 sec, followed by 40 cycles at 95°C for 5 sec, 60°C for 35 sec, and 95°C for 15 sec, 60°C for 1 min, and 95°C for 15 sec, as described previously [31]. Expression levels of* smad2* and miR-26a/b were normalized to *β-actin* or U6 small nuclear RNA (snRNA) expression. The data was analyzed by using the comparative CT method [56].

### 2.6. Statistical Analysis

All statistical analyses were performed by SPSS software version 17.0 (SPSS, Inc., Chicago, IL, USA) and the results were shown as the mean ± standard deviation (SD) of at least three biological replicates. The* p *values were determined by a 2-sided *t*-test and one-way ANOVA followed by the Tukey's post hoc test which was considered as the reference of statistically significant difference.

## 3. Results

### 3.1. Differentially Expressed miRNAs Detected in Both Growth and Selection Phases of DFs

**Figure 1:**
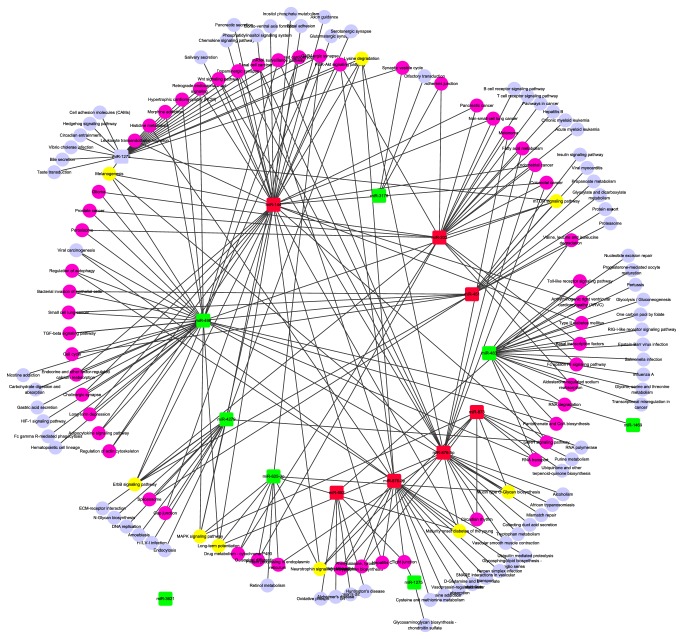
Network of the common differentially expressed miRNAs and their functionally associated signaling pathways in DFs comparing with both small healthy follicles and SFs. The red squares represent the upregulated miRNAs while green squares represent downregulated ones. The purple nodes represent pathways affected by two or three dysregulated miRNAs and the yellow nodes represent pathways coregulated by at least 4 dysregulated miRNAs. Other pale blue circles represent pathways affected by only one differentially expressed miRNA in DFs comparing with both small healthy follicles and SFs.

Follicular development is a complicated and elaborate process. Both the growth and selection of DFs are significant phases of the follicular development. A prerequisite for understanding follicular progression is acquiring knowledge of its component interactions. In order to investigate the detailed spatiotemporal miRNA profiles during follicular development from small healthy follicles to large healthy follicles, GEO repository (GSE54692) was analyzed in depth and the comparative results of different follicular groups (DFs versus small follicles and DFs versus large atretic follicles) demonstrated that there were 17 and 57 miRNAs differentially expressed (larger than or equal to twofold; adjusted* p *value ≤ 0.05) between DFs and small follicles as well as between DFs and large atretic follicles, respectively [48]. Out of them, 15 miRNAs (7 upregulated miRNAs and 8 downregulated miRNAs) were differentially expressed in DFs compared to both small follicles and large atretic follicles (Table 1). Then we used DIANA-mirPath and TargetScanHuman 7.0 to identify the target genes and affected pathways associated with these 15 differentially expressed miRNAs (S1 Table in Supplementary Material available online at https://doi.org/10.1155/2017/4585213). This network showed interaction between 7 upregulated as well as 8 downregulated miRNAs and their identified 139 signaling pathways (Figure 1). Among them, there were 29 pathways coregulated by two or three dysregulated miRNAs. 7 pathways were identified to be influenced by at least four differentially expressed miRNAs in DFs when comparing with small healthy follicles or large atretic follicles (Figure 1). The concrete miRNAs which coregulated these 7 pathways have been showed in S2 Table. For example, MAPK signaling pathway was identified as a target of miR-873-5p, miR-144-3p, miR-625-3p, miR-498, and miR-4279, while miR-3621 was a special node without connections to any other pathway since our absent cognition about its potential function. Taking into consideration that MAPK signaling pathways play a key role in follicular function, this result suggests the significance of differentially expressed miRNAs.

### 3.2. Abundance of miRNAs in GCs of DFs and SFs during Estrous Cycle

Since every step of the follicular development is important and worthy of investigation, clarifying the profile of miRNAs in the two main kinds of follicles is a burning problem due to miRNAs' regulation process on gene expression quantity which has been demonstrated by plenty of scientific evidence [57]. As reported in the relevant GEO datasets (GSE56002 and GSE55987), 6 miRNA sequencing libraries were generated based on GCs detecting in both DFs and SFs to understand the abundance and functions of miRNAs in different follicular styles. After filtering PCR primers, low-quality reads, sequences shorter than 18 bps, and empty adaptors, the mean quality reads of the biological triplicates approximated 2.4 and 3 million in the libraries of DFs and SFs, respectively. Sequence alignment of all reads which met the criteria indicated that 663,338 and 928,373 reads in DFs and SFs were mapped to the bovine reference genome, constituting 27.6% and 31.4% of the total quality reads obtained, respectively. Furthermore, 343,221 reads in DFs and 467,028 in SFs were similar to known bovine miRNAs reported in miRbase release 20 (Table 2(a)).

Based on the sequencing results, several miRNAs were commonly abundant in both datasets. Comparing the top 10 abundantly expressed miRNAs in each group, miR-26a, miR-10b, let-7 families (let-7a-5p, let-7f, and let-7i), miR-27b, and miR-191 were consistently observed in both DFs and SFs (Table 2(b)). To sum up, the network indicated that these abundantly expressed miRNAs might play key roles in maintaining the normal physiological function in DFs as well as SFs.

### 3.3. Canonical Pathways Associated with the Top 7 Abundantly Expressed miRNAs in GCs of DFs and SFs

**Figure 2:**
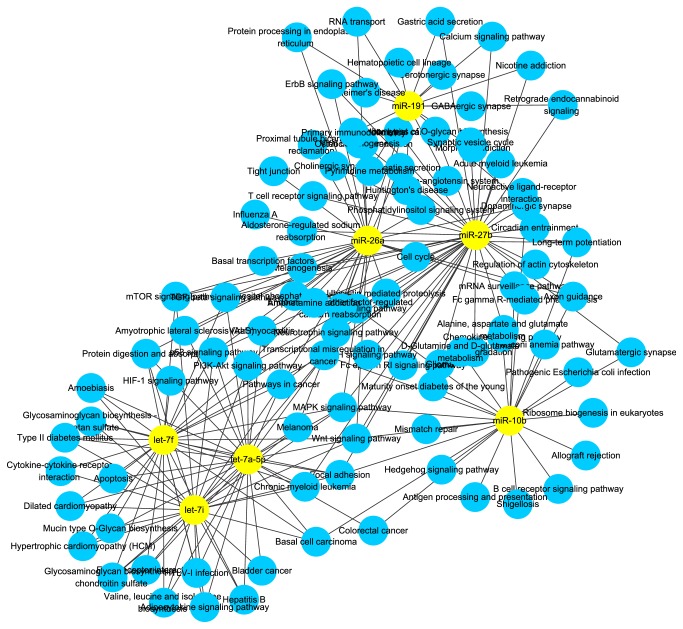
Network of abundantly expressed miRNAs and correlative pathways in both DFs and SFs. Yellow nodes represent abundantly expressed miRNAs in common while blue nodes represent the pathways affected by miRNAs.

**Figure 3:**
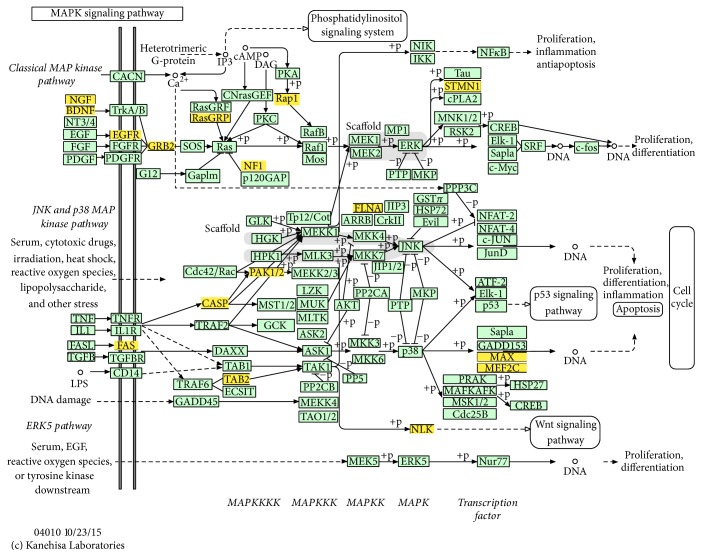
MAPK signaling pathway. This map of MAPK signaling pathway was obtained based on KEGG. The yellow boxes represent target genes which were regulated by the 7 critical miRNAs identified by previous analysis.

In order to understand the functional involvement of the 7 common miRNAs in follicular development, target genes of each miRNA were predicted and the relational canonical pathways were performed by mirPath. As a result, a total of 195 canonical pathways were affected by the predicted target genes of highly expressed miRNAs (S3 Table). There were 44 pathways regulated by at least 2 different miRNAs among which 16 pathways were coregulated by more than 3 miRNAs (Figure 2). Thereinto, MAPK is an important signaling pathway in cell proliferation and may function in granulosa cell death and follicular atresia [58]. It was comodulated by all the 7 miRNAs including miR-26a, miR-10b, let-7 families (let-7a-5p, let-7f, and let-7i), miR-27b, and miR-191. To understand the regulation mechanism of miRNAs in the MAPK signaling pathway, target genes for the synergic miRNAs were identified. Deserved to be mentioned, the let-7 families regulate the 6 genes and miR-27b targets 8 genes in MAPK pathway (Table 3). These target genes spread in the different positions of MAPK signaling to influence various functional modules and further establish interactions with other pathways such as Wnt signaling pathway (Figure 3). Therefore, it speculated that these miRNAs regulate some processes of follicular development through modulating MAPK signaling pathway.

### 3.4. Canonical Pathways Affected by Differentially Expressed miRNAs in GCs of DFs Compared with SFs

**Figure 4:**
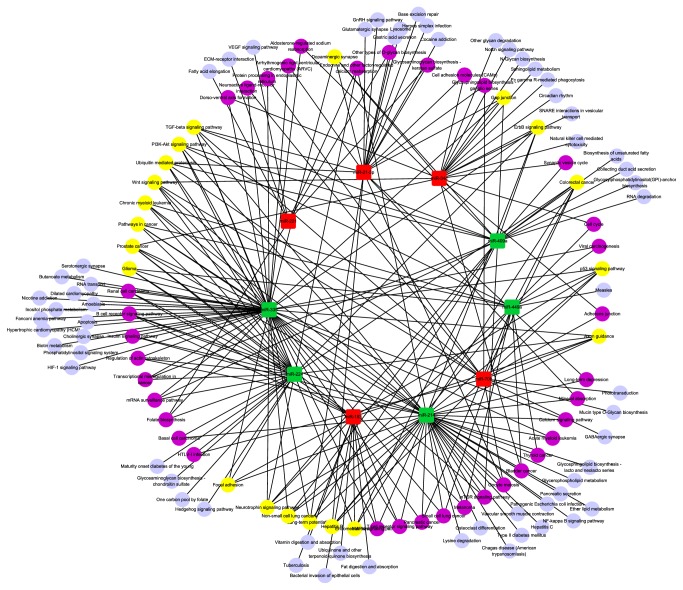
Network of signal pathways and their respective dysregulated miRNAs in DFs. The red squares represent the upregulated miRNAs while the green squares represent downregulated ones. The purple nodes represent pathways affected by two or three dysregulated miRNAs and the yellow nodes represent pathways coregulated by at least 4 dysregulated miRNAs. Other pale blue circles represent pathways affected by only one differentially expressed miRNA in DFs comparing with SFs.

**Figure 5:**
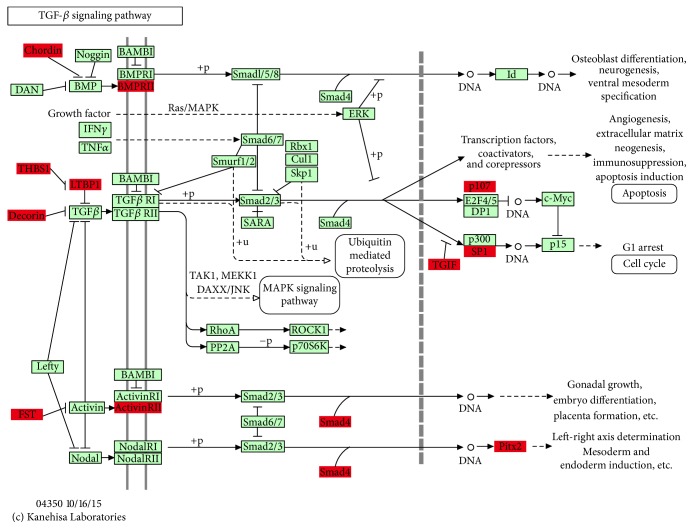
TGF-*β* signaling pathway. This map of TGF-*β* signaling pathway was based on KEGG where the red boxes represent target genes regulated by significantly differentially expressed miRNAs through predicting results.

According to the comparison of the two sequencing datasets from GEO (GSE56002 and GSE55987), there were 10 miRNAs significantly differentially expressed in GCs of DFs compared with SFs, of which 5 matured miRNAs, namely, miR-183, miR-34c, miR-708, miR-21-3p, and miR-221, were significantly upregulated in DFs while the others including miR-409a, miR-335, miR-449a, miR-214, and miR-224 were significantly lower expressed (S4 Table). The overall log2 fold change values ranged from −16.4 (miR-409a) up to 7.03 (miR-183). We also used DIANA-mirPath v2.0 software and TargetScanHuman 7.0 to identify target genes and regulatory pathways of differentially expressed miRNAs were compared and analyzed using the same method (S5 Table). There were 31 pathways coregulated by two or three dysregulated miRNAs, such as Adherens junction and PI3K-Akt signaling, respectively (Figure 4), while there were 16 pathways coregulated by at least 4 dysregulated miRNAs such as TGF-*β* signaling pathway. TGF-*β* signaling pathway has been demonstrated to play a crucial role in the local modulation of cell-cell communication [59]. Thus, TGF-*β* signaling pathway might exhibit regulatory function for human GCs in ovarian physiology. Specifically, considering that the influences of miRNAs on signaling pathways were achieved by manipulating relevant genes involved in signaling pathways, genes in the TGF-*β* signaling pathway regulated by miRNAs or in a complex miRNAs' combination were illustrated in Table 4. For example, miR-335 could regulate 7 genes, namely, DCN, FST, THBS1, RBL1, ACVR2A, LTBP1, and BMPR2, while THBS1 was also regulated by miR-708, where the two miRNAs had different regulated directions in DFs (Figure 5). Because of the synergetic regulation of several significant miRNAs, TGF-*β* signaling pathway might be closely related to follicular development. Besides, a single differentially expressed miRNA might regulate several pathways simultaneously. All of these complex relations between miRNAs and their modulating pathways thus form a functional network related to the different periods of estrous cycle.

### 3.5. GO Functional Enrichment Analysis of the Pivotal miRNAs

Based on the above findings, GO analysis seems to be meaningful with respect to the function of miRNAs on follicular development process, especially the miRNAs with high abundance and significantly differentially expressed miRNAs, which could be recognized as the pivotal miRNAs. After screening the target gene prediction of the pivotal miRNAs with the thresholds of certain *P*_CT_ and context score, 696 genes were identified as the functional target genes. By employing the DAVID software, a total of 260 GO function items were enriched. Among them, 206 were enriched in biological process (BP), 20 in cellular component (CC), and the others in molecular function (MF). The top 5 of the significant GO terms (with the smallest* p *value) in each category were shown in Table 5. Interestingly, in BP, the top 5 functions are correlative with embryonic development. It suggested that these miRNAs and target genes participated in not only follicular development but also embryonic development.

### 3.6. The miRNA-TFs Coregulatory Network in DFs and SFs

**Figure 6:**
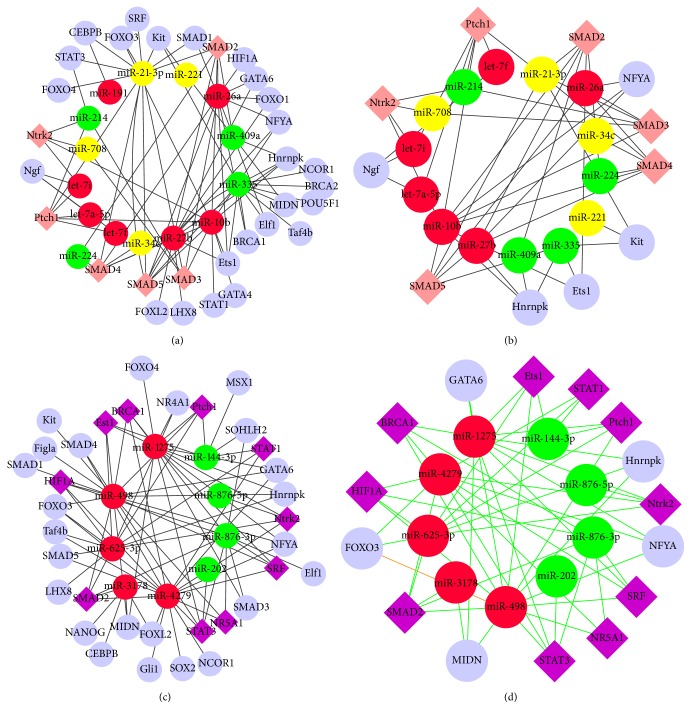
Network of miRNAs and TFs. (a) Network of TFs and pivotal miRNAs confirmed in comparison of DFs and SFs. (b) The core network shrank by extracting the hub TFs from (a). The red nodes represent abundantly expressed miRNAs both in DFs and SFs. The yellow nodes represent upregulated and the green nodes represent downregulated miRNAs. The pink rhombic nodes represent TFs affected by differentially expressed miRNAs. (c) Network of TFs and critical miRNAs confirmed in comparison of DFs versus SFs as well as DFs versus small follicles. (d) The relevant core network constructed by the relations between the hub TFs and miRNAs with correspondence to (c). The red nodes represent upregulated miRNAs and green nodes represent downregulated miRNAs. The purple rhombic nodes represent TFs affected by both differentially expressed miRNAs. The orange lines display the connections of TFs and miRNA which had evidenced basis [43–46].

Based on the hypothesis that the complexity of the eukaryotic transcriptional regulation machinery reflects a multitude of responses and that regulatory axes involving miRNAs and TFs are not isolated instances, the predicted TFs were incorporated along with the differentially expressed miRNAs in a transcriptional network. Corresponding to the total predicted genes of the pivotal miRNAs in follicular development, a total of 31 transcription factors involved in control of particular ovarian functions retrieved from published scientific reports were collected. Then the regulating network between 14 miRNAs and 30 TFs was extracted (Figure 6(a)). Some TFs, referring to SMAD2, SMAD3, SMAD4, SMAD5, and Ptch1, have been affected by the most miRNAs with respect to their highest degrees in the network. Since the critical significance of the TFs modulated by multiple miRNAs, the hub TFs recognized as carrying degree larger than 2 (S6 Table) were determined, and a shrunk miRNA-TF regulatory network only covering miRNAs and the hub TFs was subsequently generated (Figure 6(b)). Similarly, a network consisting of 35 TFs and several dysregulated miRNAs obtained from the comparison of DFs versus small healthy follicles and DFs versus SFs was constructed (Figure 6(c)) in which STAT3 and SMAD2 showed prominent performance in terms of their highest connections with miRNAs (5 miRNAs for each). Moreover, 21 TFs were regulated by downregulated miRNAs alone while only 3 TFs were regulated by upregulated miRNAs alone. Similarly, the relevant shrunk network covering the 11 hub TFs (with degree larger than 2, S7 Table) has been constructed to represent the core miRNA-TF regulatory relations (Figure 6(d)). SMAD2 might play a key role in follicular development because of its participation in both the core miRNA-TF networks as shown in Figures 6(b) and 6(d). The network of predicted TFs and miRNAs indicated the possible transcriptional regulation in follicular development.

**Figure 7:**
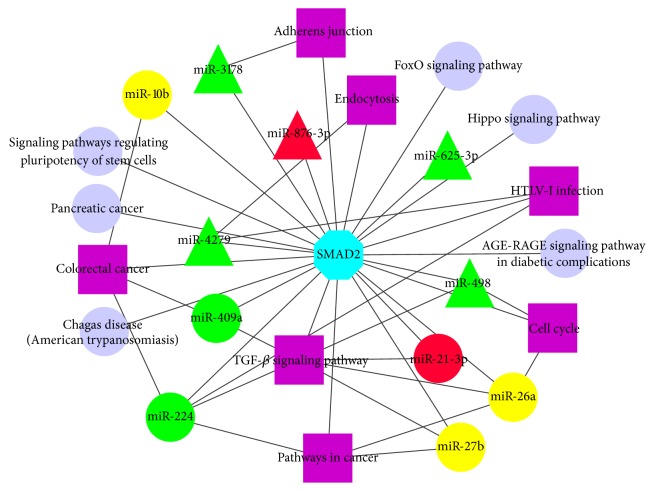
Network of SMAD2 and relevant pathways regulated by identified miRNAs. The yellow circular nodes represent highly abundant miRNAs both in DFs and SFs. Red circular nodes represent the upregulated miRNAs in DFs and the green circular nodes represent the downregulated miRNAs in DFs. The red and green triangular nodes represent up- and downregulated miRNAs in DFs when compared with either small healthy follicles or SFs, respectively. The purple square nodes represent pathways regulated by these identified miRNAs and SMAD2 simultaneously.

SMAD superfamily including SMAD1, SMAD2, SMAD3, SMAD4, and SMAD5 have been demonstrated to have functional correlations with ovarian follicular growth and selection [60–62]. SMAD2 could be regulated by all the identified important miRNAs from different detecting groups in our study. We found that TGF-*β* signaling pathway would be regulated by several miRNAs related to follicular development such as miRNA-21-3p while it also was mediated by SMAD superfamily [63]. There is a group of miRNAs that could coregulate both some signaling pathways (such as TGF-*β* signaling pathway) and SAMD2. Interestingly, in these identified pathways regulated by miRNAs, SMAD2 frequently affects the activity of these pathways as a key functional component (Figure 7). In conclusion, SMAD2 could be considered as a bridge, connecting predecessors and successors and affecting pathway activity in response to environmental signals. The result also suggested that, in ovarian follicular development, different regulatory elements functioned synergistically in networks rather than working alone.

### 3.7. The Regulatory Impact of miR-26a/b on the Expression of* smad2* in KGN Cells

**Figure 8:**
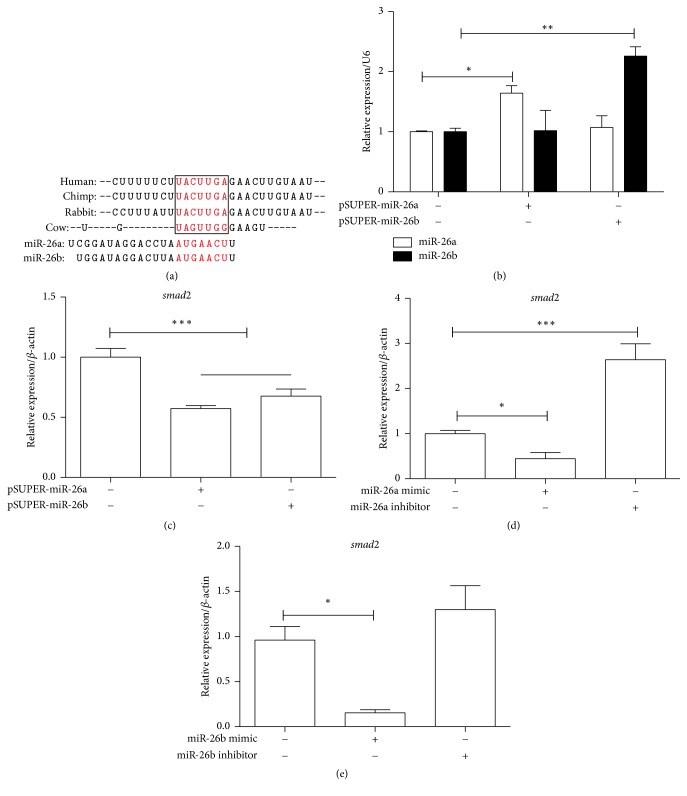
The effect of miR-26a/b on the regulating* smad2* mRNA in KGN cells. (a) A sketch map describes predicted seed sequences of miR-26a/b in the conservative 3′-UTR of* smad2* in several mammal species. (b) The transfection efficiency of overexpression vectors of miR-26a/b (pSUPER-miR-26a and pSUPER-miR-26b) was validated by RT PCR. (c, d, and e) The effects of pSUPER-miR-26a/b, miR-26a/b mimics, and miR-26a/b inhibitors on the* smad2* mRNA level were identified in KGN cells, respectively. ^*∗*^*p* < 0.05, ^*∗∗*^*p* < 0.01, and ^*∗∗∗*^*p* < 0.001; all experiments were independently repeated three times.

As the above results of this study, we deduced that some miRNAs may influence follicular development process through implementing regulation on some genes and associated signaling pathways, for instance, the TF,* smad2.* However, it was primary prediction based on the importance on its functional networks, and few evidences for the prediction were provided by experimental researches. A related research reported that miR-26b could induce apoptosis in GCs by targeting SMAD4, both directly and indirectly through USP9X, which regulated the ubiquitination of SMAD4 [63, 64]. Since miR-26b and miR-26a are highly homologous and in many species of mammals the seed sequences are also highly conservative (Figure 8(a)), we conducted the investigation of the regulatory function of the miR-26a/b on* smad2*. The miRNA overexpression vectors, miR-26a/b mimics, and miR-26a/b inhibitors were transfected into KGN cells successively. The results showed that overexpression of miR-26a/b in KGN cells (Figure 8(b)) led to the significant suppression of* smad2* (Figure 8(c)). Cells transfected with miR-26a/b mimics displayed a significantly decreased expression of* smad2*. In contrast, the miR-26a inhibitor transfected cells had an obviously increased tendency of* smad2* expression compared with NC transfected cells (Figures 8(d) and 8(e)). It suggested that miR-26a/b could regulate the expression of* smad2* in KGN cell line. However, whether miR-26a/b could directly target SMAD2 needs further research. Thus, this experiment probably provides preliminarily supportive evidence on our speculative efforts. In this context, other important regulating relations among miRNAs as well as genes and signaling pathways related to follicular development, which were identified by our analysis, may be worth studying in depth.

## 4. Discussion

Follicular development during the estrous cycle is an extraordinarily complex and synergistic process which might be regulated accurately by plenty of regulatory factors such as miRNAs and TFs. The aim of this study was to analyze the function of significant miRNAs macroscopically and associated functional networks related to follicular development via bioinformatic investigation. It is worth noting that miRNAs are largely conserved in different species of mammal. Furthermore, studies of miRNAs in ovarian tissues had confirmed the similar expression patterns in ovaries of various species, including humans [16], mice [17, 57, 65], pigs [18], sheep [66], goats [19], and cows [20, 67–69]. Through these references and related data, miRNAs which were discovered in transcriptome of bovine are also subsistent in humans and mice. Therefore, it is feasible that TargetScanHuman and DIANA, which are developed mainly for human, also could be used to predict target genes of differentially expressed miRNAs in bovine because of sequence conservation and the relevant algorithm basis. miRNA deep sequencing quantifies the relative abundance of miRNAs by their frequencies in terms of read counts. Highly abundant miRNAs have higher likelihood of higher read counts compared to miRNAs with lower abundance [70]. In GSE56002, from all reads that met the quality control criteria, 343,221 reads in DFs and 467,028 in SFs were found to be similar to known miRNAs reported in miRbase [24]. Among the detected miRNAs which appeared in both GSE56002 and GSE55987, lots of miRNAs such as isoforms of let-7 family were commonly expressed with high levels in both DFs and SFs. It implied that this kind of miRNAs might not regulate selection of DFs but maintain normal physiological functions in GCs of both DFs and SFs during the estrous cycle. The previous studies had demonstrated that the let-7 family could regulate steroidogenesis and expression of steroidogenesis-related genes [71]. In fact, steroidogenesis is a crucial biology process for maintaining normal physiology function in both DFs and SFs. Not only let-7 family but also miR-191 and miR-26a present abundant expression which could increase the proliferation of GCs [72] and regulate gonad development partially through its target on* exm2* [73]. Specifically, miR-26a showed abundant expression in both DFs and SFs, while the reads of miR-26a screened in SFs were actually more than in DFs. The results indicated that miRNA-26a might play an important role in discrepant functions between SFs and DFs. Furthermore, the canonical pathways, such as PI3K-Akt signaling pathway, MAPK signaling pathway, TGF-*β* signaling pathway, Wnt signaling pathways, and Axon guidance, which were enriched from abundant miRNAs also related to cell metabolism and signal transduction in normal follicular development. It demonstrated that the functions of these regulated pathways were of equal importance in different follicular types. Some research articles have suggested EGFR, MEF2C, and BDNF in MAPK signaling pathway were regulated by miR-27b and miR-191, respectively [74–77]. Meanwhile, it has been demonstrated that let-7 family might be involved in the estrous cycle through MAPK signaling pathway [78–80].

Abundantly expressed miRNAs might have key roles in maintaining the normal status of follicles while differentially expressed miRNAs would exhibit more regulatory functions in follicular growth, selection, and the fate of DFs and SFs. A total of 10 miRNAs were dysregulated between DFs and SFs and the result might provide valuable insights into their potential roles in folliculogenesis in a stage-dependent manner. Thus, it could be assumed that several signal pathways influenced by differentially expressed miRNAs were associated with growth and selection of follicles. Previous studies reported that increased cell apoptosis would be observed in GCs transfected with the miR-21 inhibitor. Furthermore, the critical roles of miR-21 in regulating follicular development and preventing GCs apoptosis in DFs had also been verified [35], while miR-183, upregulated in DFs, might induce cell apoptosis [72]. It suggested that in follicular development, multiple miRNAs, pathways, or TFs work simultaneously, though they might show some opposite effects. In addition, SMAD4 which played a key role in TGF-*β* pathway would be targeted by miR-224 [31], while miR-214/199a and miR-335 could affect TGF-*β* pathway in different manners [81, 82]. TGF-*β* superfamily members have been implicated in regulating GC proliferation [83] and terminal differentiation [84] that are critical for normal ovarian follicular development. Furthermore, by GO functional enrichment, we found that the miRNA target genes played key role in follicular development, which can also effect the embryonic development. It is well known that follicular development and embryonic development are both two important biological processes for reproduction. The roles of some miRNA target genes in both these processes were probably similar. For instance, the genes in Wnt signaling pathway were regulated by same miRNAs in both follicular development and embryonic development [46].

miRNAs participate in follicular development not only in one single manner; there is another primary mode to command maturation of follicles related to critical transcription factors. Similar to the significance of pathway regulation, TFs also could influence the regulatory network in mediating follicular development. According to the bioinformatic analysis, SMAD2 might be coregulated by miR-27b, miR-10b, and miR-26a which were expressed abundantly in both follicle types. miR-21 and miR-409a with crosscurrent in DFs also could regulate* smad2*. Interestingly, among these miRNAs, miR-21 was demonstrated to be regulated via SMAD2/3 signaling [85]. It suggested that SMAD2 interacted with some miRNAs and played key roles in follicular development. miR-26a and miR-26b are highly homologous and the seed regions are highly conservative in several species. In our study, we concluded that miR-26a/b could decrease the mRNA expression of* smad2* through RT PCR results. Nevertheless, the detailed regulatory mechanisms need to be further studied. Because of the abundance of miR-26, both in DFs and in SFs,* smad2* expression may be in a low level and the correlation pathways may be in an inactive status. Moreover,* smad2* was also coregulated by miR-498, miR-3178, miR-4279, and miR-625-3p, which were downregulated, and miR-876-3p that was upregulated in DFs compared with both small follicles and SFs. Although the complex correlation of SMAD2 and significant miRNAs made this TF a hub in the whole gene regulatory network of follicular development, deficient evidence about the functional relations of SMAD2 and miRNAs was obviously scarce to confirm this description. The functions of SMAD2-miRNAs network in follicular development need further study. Besides SMAD2, other members of SMAD superfamily such as SMAD3 or SMAD4 were also demonstrated to be associated with miRNAs and signaling pathways [31, 86], especially TGF-*β* signaling pathway which had illustrated affecting ovary development [87]. For example, miR-21 could regulate the expression of SMAD7 and TGF-*β*1 [88]. The accurately regulatory network composed of important miRNAs and hub TFs would provide a valuable perspective for understanding the formation of DFs during estrous cycle.

Precisely because of the miscellaneous connection among miRNAs, signaling pathways, and TFs, the regulatory network in final maturation of follicle and preparation for the subsequent follicle-luteal transition would be more thorough. Our coregulatory network will help to draw the dynamic changes of miRNAs and associated regulatory modules across a wide range of follicular developmental stages. However, there were also some deficiencies in this study. The work was not based on the relationship of miRNAs and hormones which play a key role in the whole estrous cycle. Several miRNAs were demonstrated to be regulated by hormones, such as luteinizing hormone (LH), hCG (human chorionic gonadotropin), and follicle stimulating hormone (FSH) [31, 35, 43, 89] which displayed important controls on ovarian functions. It suggested that miRNAs may regulate ovarian functions associated with changes of hormones content. Moreover, although the significant miRNAs and their influencing pathways or TFs were identified, the functions of differentially expressed miRNAs in follicular development were still unclear. Despite these limitations, this study still predicted possible details about molecular regulation network and identified probable key regulators in the process of follicular development, which provided a novel idea to understand the regulation of follicular development deeply, as well as diagnosis and treatment for infertility.

## 5. Conclusions

In this study, via bioinformatic analysis, we concluded that during the growth and selections of DFs lots of miRNAs in GCs which are abundantly expressed (let-7) or dysregulated (miR-21-3p) take part in biology processes by forming networks with TFs (such as SMAD superfamily) and pathways (TGF-*β* signaling pathway). By GO functional enrichment analysis of the pivotal miRNAs target genes, 260 GO function items in BP, CC, and MF were enriched. SMAD2, regulated by miR-26a/b, has been demonstrated by RT PCR as one of these kinds of TFs may play a key role in several pathways as a participator. It suggested that miR-26a/b-SMAD2-TGF-*β* signaling pathway might play a significant role in follicular development as an axis. Having said that, we wish to emphasize that the functions of other pivotal miRNAs, TFs, and pathways need more studies at macro level.

## Supplementary Material

The Supplementary Material include 7 tables. The Supplementary Table 1 list the miRNAs down-regulated in DFs and associated pathways compared with small health follicles and large atretic follicles. The Supplementary Table 2 list the pathways regulated by both up- and down- regulated miRNAs in DFs compared with small health follicles and large atretic follicles. The Supplementary Table 3 list the abundant miRNAs both in DFs and SFs and associated pathways. The Supplementary Table 4 list the deregulated miRNAs in Both Two GSEdatabases. The Supplementary Table 5 list the up and down-regulated miRNAs in DFs and associated pathways. The Supplementary Table 6 list the analyses of every note in miRNA-TF network of DFs compared with SFs. The Supplementary Table 7 list the analyses of every note in miRNA-TF network of DFs compared with both small health follicles and large atretic follicles.

## Figures and Tables

**Table 1:** Fold changes of differentially expressed miRNAs in DFs compared with both small healthy follicles and SFs.

	miRNA ID	DFs versus SHFs		DFs versus SFs	
		Fold change	Adj.* p *value	Fold change	Adj. *p *value
Downregulated miRNAs	miR-3178	−3.60	0.002	−3.36	0.001
	miR-1275	−2.29	0.020	−2.28	0.003
	miR-625-3p	−2.21	0.006	−4.94	0.001
	miR-3621	−2.17	0.002	−2.21	0.001
	miR-483-3p	−2.17	0.002	−3.64	0.001
	miR-1469	−2.15	0.014	−5.11	0.001
	miR-498	−2.14	0.003	−3.69	0.001
	miR-4279	−2.05	0.012	−2.96	0.001

Upregulated miRNAs	miR-202	4.18	0.002	9.98	0.001
	miR-876-5p	3.79	0.002	5.99	0.001
	has-miR-876-3p	3.09	0.002	3.92	0.001
	miR-873-5p	2.70	0.003	3.53	0.001
	miR-451a	2.65	0.014	2.49	0.031
	miR-144-3p	2.35	0.009	2.02	0.032
	miR-652-3p	2.13	0.003	3.50	0.001

**(a):** 

Group	Sample	Number of mapped reads	Reads aligned to known miRNAs	Arithmetic mean of mapped reads	Arithmetic mean of known miRNAs	Ratio of known miRNAs
Dominant follicles	DFs1	599377	345689	663338.7	343221.7	0.5174
	DFs2	392924	255260			
	DFs3	997715	428716			

Subordinate follicles	SFs1	861596	459794	928373	467028	0.5031
	SFs2	939835	520377			
	SFs3	983688	420913			

**(b):** 

miRNA	DFs			miRNA	SFs		
	Reads1	Reads2	Reads3		Reads1	Reads2	Reads3
*miR-26a*	48999	53635	42285	*miR-26a*	77730.33	37599	61123
*miR-10b*	28168.67	46558	14742	*miR-10b*	62390	37494	63322
miR-202	12209	4473	<1000	miR-92a	13653.33	8852	11115
*let-7a-5p*	10838.33	14314	10417	*let-7f*	13331	11065	11611
*let-7f*	9595.33	14697	8616	*miR-27b*	13194.67	12922	11789
miR-22-3p	8710.33	3626	5359	miR-99b	12241.33	18612	15768
*let-7i*	8695.67	13802	7269	*let-7a-5p*	11003.67	10133	10026
miR-21-5p	8695.33	<1000	36422	*let-7i*	9734.67	8944	11203
*miR-27b*	8476.67	15893	20250	*miR-191*	8563	8073	9995
*miR-191*	7700.33	15491	5147	miR-143	8397.67	3736	3906

Oblique font represents identical miRNAs both in DFs and SFs.

Reads1: the data were acquired from GSE56002.

Reads2: the data were acquired from Day 3 in GSE55987.

Reads3: the data were acquired from Day 7 in GSE55987.

**Table 3:** Target genes regulated by top 7 abundant miRNAs involved in MAPK signaling pathway.

miRNA ID	Target genes
miR-26a	TAB2, NLK, MEF2C, PAK1
miR-10b	MAX, MEF2C, RAP1A
let-7a-5p	FLNA, NGF, FAS, MEF2C, CASP3, RASGRP1
let-7f	FLNA, NGF, FAS, MEF2C, CASP3, RASGRP1
let-7i	FLNA, NGF, FAS, MEF2C, CASP3, RASGRP1
miR-27b	EGFR, TAB2, NLK, NF1, FAS, STMN1, MEF2C, GRB2
miR-191	BDNF

**Table 4:** Target genes regulated by differentially expressed miRNAs in TGF-*β* signaling pathway.

miRNA ID	Target genes
miR-708	THBS1
miR-21-3p	TGIF1, SP1
miR-409a	PITX2, BMPR2
miR-335	DCN, FST, THBS1, RBL1, ACVR2A, LTBP1, BMPR2
miR-224	SMAD4, LTBP1, BMPR2
miR-214	CHRD, ACVR2A

**Table 5:** The top 5 GO terms enriched by the pivotal miRNAs.

Category^1^	Term	Description	Count^2^	*p *value^3^
BP	GO:0048598	Embryonic morphogenesis	34	1.7*E* − 10
	GO:0035113	Embryonic appendage morphogenesis	16	3.8*E* − 8
	GO:0030326	Embryonic limb morphogenesis	16	3.8*E* − 8
	GO:0043009	Chordata embryonic development	31	6.3*E* − 8
	GO:0009792	Embryonic development ending in birth or egg hatching	31	7.7*E* − 8

CC	GO:0005667	Transcription factor complex	19	2.00*E* − 05
	GO:0044451	Nucleoplasm part	32	1.40*E* − 04
	GO:0005654	Nucleoplasm	44	1.70*E* − 04
	GO:0005626	Insoluble fraction	41	4.50*E* − 04
	GO:0005624	Membrane fraction	39	8.20*E* − 04

MF	GO:0003700	Transcription factor activity	53	2.60*E* − 05
	GO:0016563	Transcription activator activity	28	9.60*E* − 05
	GO:0030528	Transcription regulator activity	71	9.90*E* − 05
	GO:0043565	Sequence-specific DNA binding	35	2.90*E* − 04
	GO:0003705	RNA polymerase II transcription factor activity, enhancer binding	7	9.40*E* − 04

*Notes*. ^1^GO function category; ^2^the number of target genes involved in the GO terms. ^3^*p *values have been adjusted using the Benjamini–Hochberg method.

BP, biological process; CC, cellular component; MF, molecular function; GO: Gene Ontology.
